# Effectiveness and safety of direct oral anticoagulants versus vitamin K antagonists in atrial fibrillation patients with liver disease: a systematic review and meta-analysis

**DOI:** 10.3389/fphar.2025.1620394

**Published:** 2025-07-14

**Authors:** Qiang Zhou, Xiang Liu, Shuyu Liu, Zhichun Gu, Yanzi Wu, Yuansu Yang, Yingying Tao, Meng Wei

**Affiliations:** ^1^ Department of Clinical Pharmacy, Jinling Hospital, Medical School of Nanjing University, Nanjing, China; ^2^ College of Traditional Chinese Medicine, Jiangsu College of Nursing, Huaian, China; ^3^ Department of Cardiology, Jinling Hospital, Medical School of Nanjing University, Nanjing, China; ^4^ Department of Pharmacy, Renji Hospital, School of Medicine, Shanghai Jiao Tong University, Shanghai, China; ^5^ Department of Research and Training, Jinling Hospital, Medical School of Nanjing University, Nanjing, China

**Keywords:** atrial fibrillation, liver disease, direct oral anticoagulants, vitamin K antagonists, anticoagulants, meta-analysis

## Abstract

**Introduction:**

Patients with atrial fibrillation (AF) and liver disease, particularly cirrhosis, are frequently excluded from anticoagulation trials, leaving the optimal therapeutic strategy uncertain.

**Methods:**

This study aimed to compare the effectiveness and safety of direct oral anticoagulants (DOACs) and vitamin K antagonists (VKAs) in patients with AF and liver disease. We systematically searched the PubMed, Cochrane Library, Medline, and Embase databases for relevant studies published up to November 2024.

**Results:**

Fourteen studies, involving 44,848 participants, were included. Compared to VKAs, DOACs were associated with significantly lower risks of major bleeding (risk ratio [RR]: 0.64, 95% confidence interval [CI]: 0.55–0.75; P < 0.0001), intracranial bleeding (RR: 0.43, 95% CI: 0.33–0.56; P < 0.0001), gastrointestinal (GI) bleeding (RR: 0.72, 95% CI: 0.59–0.89; P = 0.002), and all-cause mortality (RR: 0.83, 95% CI: 0.70–0.98; P = 0.03). No significant difference was observed in ischemic stroke/systemic embolism (RR: 0.77, 95% CI: 0.52–1.13; P = 0.19). In patients with cirrhosis, DOACs were similarly superior for major bleeding, GI bleeding, and intracranial bleeding. Subgroup analyses revealed that apixaban demonstrated a more favorable safety profile than rivaroxaban in patients with liver disease, whereas both agents showed comparable effectiveness and safety in cirrhotic patients.

**Conclusion:**

DOACs are safer and equally effective alternatives to VKAs in patients with AF and liver disease, including those with cirrhosis. In patients with liver disease, apixaban may offer additional safety benefits compared with rivaroxaban. However, in patients with cirrhosis, the effectiveness and safety profiles of the two drugs are similar.

**Systematic review registration:**

https://www.crd.york.ac.uk/PROSPERO/view/CRD42024623387

## 1 Introduction

Atrial fibrillation (AF) is one of the most common arrhythmias in older patients, affecting nearly 33 million individuals worldwide (Benjamin et al., 1998). Patients with AF have a 1.5-fold higher risk of mortality and 2.5-fold higher risk of stroke than those without AF (Go et al., 2001). Long-term anticoagulant therapy is essential for reducing the risk of thromboembolic events in these patients. Liver disease, caused by hepatitis B or C virus infection, alcohol-related liver disease, and metabolic dysfunction-associated fatty liver disease, leads to hepatic impairment and abnormal coagulation, contributing to a global public health burden (Asrani et al., 2019; Xiao et al., 2025). Particularly in patients with cirrhosis, coagulation balance is often severely disrupted, elevating the risk of both venous thromboembolism (VTE) and bleeding (Tripodi and Mannucci, 2011). Therefore, clinical guidelines and expert consensus recommend anticoagulation in patients with AF and liver disease to prevent ischemic stroke or systemic embolism (Joglar et al., 2023; Husted et al., 2014).

The most commonly used oral anticoagulants include vitamin K antagonists (VKAs) and direct oral anticoagulants (DOACs). DOACs are favored over VKAs owing to their predictable pharmacokinetics, fewer food and drug interactions, and the absence of a need for regular therapeutic drug monitoring (Kirchhof et al., 2016; Chen et al., 2020). Although the safety and effectiveness of DOACs have been established in patients with AF and cancer or chronic kidney disease, their use in patients with liver disease remains controversial because such patients are frequently excluded from randomized controlled trials (RCTs) (Khorana et al., 2017; Jones et al., 2024; Schrag et al., 2023; Hydes et al., 2023). Previous meta-analyses have suggested that DOACs may be effective and safe alternatives to VKAs in patients with AF and liver disease (Chen et al., 2022). However, conflicting findings have been reported. Mort et al. reported that the risk of spontaneous bleeding was elevated in patients with liver disease receiving DOACs (Mort et al., 2021), while Song et al. found increased readmission rates associated with DOAC use compared to warfarin (Song et al., 2024). Moreover, most studies have neither compared individual DOACs nor evaluated the effects of different dosage regimens.

Given the complex balance between bleeding and thrombotic risks in patients with AF and liver disease, further evidence is needed to guide clinical decision-making (Senzolo and Garcia-Pagan, 2023). A recent meta-analysis by Miranda Maria et al. (2025) specifically examined patients with both AF and liver cirrhosis and found that DOACs were associated with lower risks of major bleeding, gastrointestinal (GI) bleeding, and all-cause mortality than VKAs, with no significant difference in thromboembolic events. However, that study focused exclusively on patients with cirrhosis and did not perform subgroup analyses by geographic region or follow-up duration, nor did it assess differences among individual DOACs or compare different dosage regimens. Additionally, their study did not apply the GRADE (Grading of Recommendations, Assessment, Development, and Evaluation) framework to assess the certainty of evidence (Miranda Maria et al., 2025).

To address these limitations, we conducted a comprehensive systematic review and meta-analysis that incorporated real-world data from studies on patients with AF and liver disease. In addition to evaluating the effectiveness and safety of DOACs versus VKAs, we performed subgroup analyses by geographic region, follow-up duration, DOAC type (apixaban vs rivaroxaban), and dose (standard vs. low-dose). We also applied the GRADE framework to assess the quality of the evidence. These enhancements aim to provide more detailed and clinically actionable guidance on anticoagulation strategies for this high-risk patient population.

## 2 Methods

This systematic review and meta-analysis were conducted in accordance with the Preferred Reporting Items for Systematic Reviews and Meta-Analyses (PRISMA) guidelines (Supplementary Table S1) (Schulman and Kearon, 2005). The study was prospectively registered with PROSPERO (registration ID CRD42024623387).

### 2.1 Data sources and search strategy

We systematically searched PubMed, Embase, MEDLINE, and the Cochrane Library for eligible studies published up to November 2024, with no language restrictions. The search strategy was as follows (atrial fibrillation OR non-valvular atrial fibrillation OR AF OR NVAF) AND (liver disease OR impaired liver function OR hepatic disease OR cirrhosis OR cirrhotic) AND (DOAC OR NOAC OR direct oral anticoagulant OR new oral anticoagulant OR non-vitamin K antagonist oral anticoagulant OR rivaroxaban OR apixaban OR dabigatran OR edoxaban) AND (VKA OR vitamin K antagonist OR warfarin). Full details of search terms were presented in Supplementary Table S2. Two reviewers (Q.Z. and S.Y.L.) independently screened the literature and extracted data. Discrepancies were resolved by discussion or consultation with a third reviewer (Y.Z.W.).

### 2.2 Inclusion and exclusion criteria

Studies were included based on the following PICO criteria: (1) population, patients with AF and liver disease; (2) intervention, DOAC therapy; (3) comparator, VKA therapy; and (4) outcomes, bleeding or thrombotic events or all-cause mortality. We excluded: (1) animal studies; (2) case reports; (3) case series; (4) review articles; (5) systematic reviews or meta-analyses; (6) studies with duplicate data; (7) single-arm studies; (8) studies with unavailable outcomes; (9) studies not involving patients with AF or liver disease; and (10) studies where DOACs were not used as anticoagulants.

### 2.3 Outcomes

Our outcomes of interest were ischemic stroke/systemic embolism (IS/SE), major bleeding, all-cause mortality, intracranial bleeding, GI bleeding. Major bleeding was defined according to the International Society on Thrombosis and Hemostasis (ISTH) (Moher et al., 2009) or the International Classification of Diseases, 9th and 10th revisions (ICD-9/ICD-10).

### 2.4 Data extraction

From each eligible study, we extracted the first author, year of publication, country or region, study population, percentage of female participants, study design, sample size, outcomes, mean age, hypertension, diabetes, combined antiplatelet therapy (APT), non steroidal anti-inflammatory drugs (NSAIDs), proton pump inhibitor/H2 receptor antagonist (PPI/H2RA), Child-Pugh score, CHA_2_DS_2_-VASc score, HAS-BLED score, follow-up duration, and the adjustment methods used for outcome comparisons.

### 2.5 Quality assessment and the certainty of evidence

Two reviewers (Q. Z. and S. Y. L.) independently assessed the methodological quality of included studies. RCTs were evaluated using the Cochrane Risk of Bias 2 (ROB 2.0) tool across five domains: randomization process, deviations from intended interventions, missing outcome data, measurement of outcomes, and selection of the reported results (Higgins et al., 2011). Non-randomized studies were assessed using the Risk Of Bias in Non-randomized Studies of Interventions (ROBINS-I) tool, which evaluates seven domains: confounding, selection of participants, classification of interventions, deviations from intended interventions, missing data, measurement of outcomes, and selection of reported results. Each domain is graded as low, moderate, serious, or no information (Sterne et al., 2016). The certainty of evidence for each outcome was rated using the GRADE approach (Atkins et al., 2004).

### 2.6 Statistical analysis

We used forest plots to measure clinical outcome events, and used a random effects model to calculate risk ratios (RR) and associated 95% confidence intervals (95% CI). Subgroup analyses were conducted using DOAC dosage (standard vs. low dose), DOAC type (apixaban vs. rivaroxaban), region (Americas, Asia, and Europe), and follow-up duration (≤12 months vs. >12 months). The standard doses included rivaroxaban 20 mg once daily, dabigatran 150 mg twice daily, and apixaban 5 mg twice daily; Low doses of rivaroxaban 10–15 mg once daily, dabigatran 110 mg twice daily, and apixaban 2.5 mg twice daily (Steffel J et al., 2021).

Sensitivity analyses were conducted by sequentially removing individual studies and excluding low-quality ones. To evaluate robustness, the results were calculated using hazard ratios (HR)/adjusted hazard ratios (aHR) and 95% CIs when available. Publication bias was assessed visually using funnel plots and the Egger’s test. All statistical analyses were performed using the Review Manager (RevMan version 5.4, Cochrane Collaboration) and Stata (version 18.0, StataCorp). Statistical significance was defined as a two-sided p-value of <0.05.

## 3 Results

### 3.1 Study selection and baseline characteristics

**FIGURE 1:**
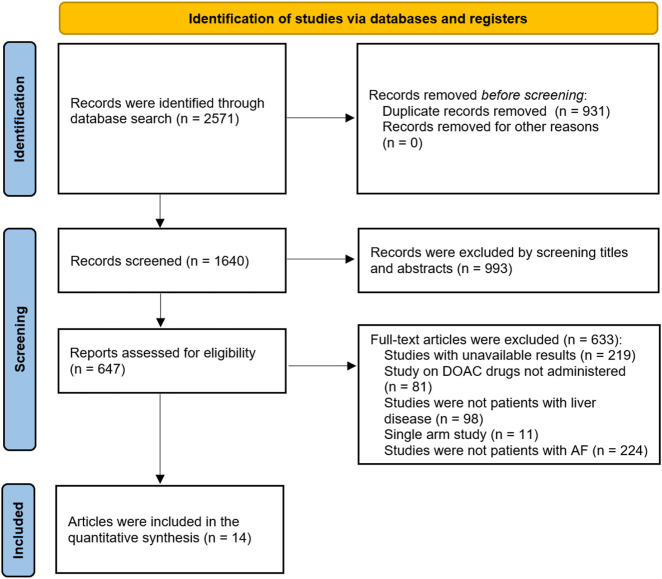
Flowchart showing the process of literature screening.

A total of 2,571 articles were initially retrieved through a systematic search. After removing duplicates and screening the titles, abstracts, and full texts, 14 studies met the eligibility criteria and were included in the meta-analysis. The PRISMA flow diagram illustrating the study selection process is shown in Figure 1 (Song et al., 2024; Chou et al., 2024; Douros et al., 2024; Simon et al., 2024; Lawal et al., 2023; Baylo et al., 2023; Yoo et al., 2022; Serper et al., 2021; Lee et al., 2019a; Qamar et al., 2019; Lee et al., 2019b; Goriacko and Veltri, 2018; Pastori et al., 2018; Wang et al., 2018).

These 14 studies involved 44,848 patients with atrial fibrillation and liver disease, including 27,807 treated with DOACs and 17,041 treated with VKAs. Of the included studies, 12 (Song et al., 2024; Chou et al., 2024; Douros et al., 2024; Simon et al., 2024; Lawal et al., 2023; Yoo et al., 2022; Serper et al., 2021; Lee et al., 2019a; Lee et al., 2019b; Goriacko and Veltri, 2018; Pastori et al., 2018; Wang et al., 2018) were cohort studies, one (Baylo et al., 2023) RCT, and one (Qamar et al., 2019) *post hoc* analysis of RCT. Five studies (Chou et al., 2024; Yoo et al., 2022; Lee et al., 2019a; Lee et al., 2019b; Wang et al., 2018) were conducted in Asian populations, three (Douros et al., 2024; Baylo et al., 2023; Pastori et al., 2018) in European populations, and seven (Song et al., 2024; Douros et al., 2024; Simon et al., 2024; Lawal et al., 2023; Serper et al., 2021; Qamar et al., 2019; Goriacko and Veltri, 2018) in American populations. One study (Douros et al., 2024) included both American and European participants. The follow-up duration across the studies ranged from 0.25 to 5.6 years. The baseline characteristics of the included studies are presented in Table 1 and Supplementary Table S3.

**TABLE 1:** Baseline characteristics of participants included in the study.

Author	Country or region	Patient population	Study design	DOAC group; n	VKA group, n	Outcomes	Follow up (year)
Chou et al. (2024)	Taiwan	Liver cirrhosis-related disease: alcoholism (16.5%); HBV infection (32.0%); HCV infection (31.8%); Nonalcoholic fatty liver disease (9.6%)	Retrospective cohort study	Dabigatran, rivaroxaban, edoxaban,and apixaban; 478	Warfarin, 237	①②③④⑤	2.6, 3.2
Douros et al. (2024)	United Kingdom and Canada	Types of liver disease: fatty liver (48.1%); cirrhosis (22.6%); alcoholic liver disease (12.1%); failure/coma (7.1%); cancer (6.7%); infection (5.1%); transplant (0.9%); other (36.2%)	Retrospective cohort study	Dabigatran, rivaroxaban, edoxaban,and apixaban; 8,815	VKAs, 3,696	②③④⑤	NA
Simon et al. (2024)	United States	Cause of cirrhosis: MASLD or MASH (72.8%); alcohol-related liver disease (26.4%); chronic viral hepatitis (13.9%); other/unspecified (13.2%)	Retrospective cohort study	Apixaban; 2,852	Warfarin, 2,852	①②③④⑤	Apixaban: 0.27, warfarin: 0.28
Simon et al. (2024)	United States	Cause of cirrhosis: MASLD or MASH (69.2%); alcohol-related liver disease (25.7%); chronic viral hepatitis (16.5%); other/unspecified (13.6%)	Retrospective cohort study	Apixaban; 2,785	Rivaroxaban, 2,785	①②③④⑤	Apixaban: 0.28, rivaroxaban: 0.24
Song et al. (2024)	United States	Cirrhosis: diagnosis of liver cirrhosis based on ICD code	Retrospective cohort study	Dabigatran, rivaroxaban, edoxabanand apixaban; 251	Warfarin, 98	③	5.2
Lawal et al. (2023)	United States	Chronic liver disease etiologies: NAFLD/NASH (30.7%); cirrhosis (28.8%); viral hepatitis (10.9%); liver cancer (1.5%); liver failure (5.1%); alcoholic liver disease (12.1%); liver diseases of genetic causes (4.4%); liver diseases of autoimmune causes (4.4%); Budd-Chiari disease (0.1%); liver transplantation (0.8%); secondary or unspecified biliary cirrhosis (0.7%); others (1.3%)	Retrospective cohort study	Dabigatran, rivaroxaban, edoxaban,and apixaban; 5,788	Warfarin, 4,421	①②③④	Approximately 0.57, 1
Baylo et al. (2023)	Ukraine	Etiology of liver cirrhosis: alcohol (42.9%); HCV (8.9%); HBV (8.9%); NAFLD (28.6%); others (10.7%)	RCT	Dabigatran (110 mg/bid); 30	Warfarin, 26	①②	0.25
Yoo et al. (2022)	Korea	Etiology of cirrhosis: alcoholic liver disease (26.9%); hepatitis B virus infection (34%); hepatitis C virus infection (10.5%); others (28.6%)	Retrospective cohort study	Dabigatran, rivaroxaban, edoxaban,and apixaban; 128	Warfarin, 110	①②	5.6
Serper et al. (2021)	United States	Etiology of cirrhosis: HCV/alcohol (72.4%); NAFLD/NASH (18%); other (9.6%)	Retrospective cohort study	Factor Xa (apixaban, betrixaban, endoxaban, and rivaroxaban) or thrombin (dabigatran); 201	Warfarin, 614	④⑤	4.6
Lee et al. (2019a)	Taiwan	Cirrhosis: including alcoholic or non-alcoholic liver cirrhosis	Retrospective cohort study	Dabigatran (110 mg/bid), rivaroxaban (10–15 mg/qd), and apixaban (2.5 mg/bid); 1,397	Warfarin, 946	①②④⑤	1.13, 1.30
Qamar et al. (2019)	United States	Liver disease: NAFLD (30.1%); baseline AST/ALT >2x ULN only (12.5%); alcohol (4.2%); viral hepatitis (19.8%); cirrhosis (1.8%); congestive hepatopathy (1.7%); others (29.6%)	Post hoc analysis of RCT	Edoxaban (30 or 60 mg/qd); 718	Warfarin, 365	①②③④⑤	2.8
Lee et al. (2019b)	Korea	Types of liver diseases: viral hepatitis (9.1%); alcoholic livre disease (7.7%); toxic liver disease (4.6%); hepatic failure (1.3%); chronic hepatitis (9.3%); liver fibrosis and cirrhosis (1.6%); other inflammatory liver disease (6.7%); other liver disease (59.1%); liver disease in diseases classified elsewhere (0.6%)	Retrospective cohort study	Dabigatran, rivaroxaban, edoxaban,and apixaban; 3,115	Warfarin, 1827	①②③④⑤	1.2
Goriacko and Veltri (2018)	United States	Etiology of chronic liver disease: alcohol (15.5%); viral (2.1%); NASH (40.8%); other (41.6%)	Retrospective cohort study	Dabigatran, rivaroxaban, and apixaban; 75	Warfarin, 158	③	1.7
Pastori et al. (2018)	United Kingdom	Advanced liver fibrosis	Prospective cohort study	Dabigatran, rivaroxaban, edoxaban,and apixaban; 1,033	VKAs, 1,297	②④⑤	2.1
Wang et al. (2018)	Taiwan	Impaired liver function: serum AST or ALT >2-fold the upper limit of normal or total bilirubin >1.5-fold the upper limit of normal	Retrospective cohort study	Dabigatran, rivaroxaban, edoxaban,and apixaban; 342	Warfarin, 394	①②③④	1

NA, not available; AF, atrial fibrillation; DOAC, direct oral anticoagulant; VKA, vitamin K antagonist; RCT, randomized controlled trial; MASH, metabolic dysfunction-associated steatohepatitis; MASLD, metabolic dysfunction-associated steatotic liver disease; NAFLD, non-alcoholic fatty liver disease; NASH, non-alcoholic steatohepatitis; HCV, hepatitis C virus; AST, aspartate aminotransferase; ALT, alaninet amino ransferase; ICD, International Classification of Diseases; BID, bis in die; QD, quaque die; IS, ischemic stroke; SE, systemic embolism; ①: IS/SE; ②: Major bleeding; ③: All-cause mortality; ④: Gastrointestinal bleeding; ⑤: Intracranial hemorrhage.

### 3.2 Quality assessment

The quality of the included studies was assessed using ROB 2.0 for RCTs and ROBINS-I for observational studies. A single RCT (Baylo A et al., 2023) was considered to have a low overall risk of bias (Supplementary Figure S1). Among the 13 non-randomized studies, four were rated as having a serious overall risk of bias (Song et al., 2024; Serper et al., 2021; Goriacko and Veltri, 2018; Pastori et al., 2018), seven as moderate (Chou et al., 2024; Douros et al., 2024; Lawal et al., 2023; Yoo et al., 2022; Lee et al., 2019a; Lee et al., 2019b; Wang et al., 2018), and two as low (Simon et al., 2024; Qamar et al., 2019) (Supplementary Figure S2).

The certainty of the evidence was rated using the GRADE framework. In patients with liver disease, the evidence for intracranial bleeding was graded as high quality, whereas evidence for major bleeding and gastrointestinal (GI) bleeding was of low quality. The evidence for IS/SE and intracranial bleeding was rated very low. In patients with liver cirrhosis, the certainty of evidence was moderate for IS/SE, major bleeding, and GI bleeding and low for all-cause mortality and intracranial bleeding (Supplementary Tables S4 and S5).

### 3.3 Outcomes in patients with liver disease

**FIGURE 2:**
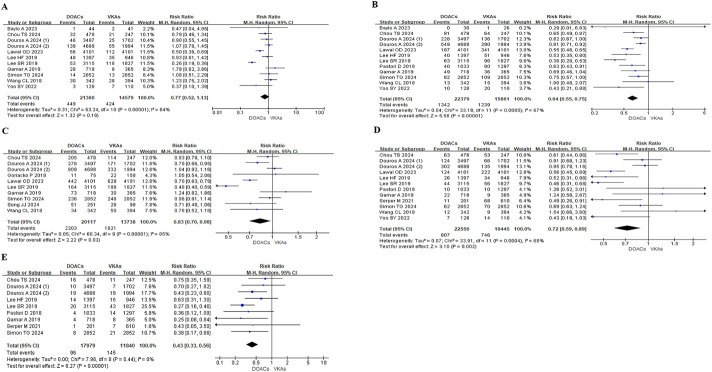
Meta-analysis of outcomes in patients with liver disease. DOAC, direct oral anticoagulant; VKA, vitamin K antagonist; IS, ischemic stroke; SE, systemic embolism; RR, risk ratio; CI, confidence interval. **(A)**. IS/SE **(B)**. Major bleeding **(C)**. All-cause mortality **(D)**. Gastrointestinal bleeding **(E)**. Intracranial bleeding.

Among patients with liver disease and AF, DOACs were associated with significantly lower risks of major bleeding (RR: 0.64, 95% CI: 0.55–0.75; P < 0.0001), GI bleeding (RR: 0.72, 95% CI: 0.59–0.89; P = 0.002), intracranial bleeding (RR: 0.43, 95% CI: 0.33–0.56; P < 0.0001), and all-cause mortality (RR: 0.83, 95% CI: 0.70–0.98; P = 0.03) compared to VKAs. No significant difference was observed in the risk of IS/SE between the two groups (RR: 0.77, 95% CI: 0.52–1.13, P = 0.19) (Figure 2).

### 3.4 Outcomes in patients with liver cirrhosis

**FIGURE 3:**
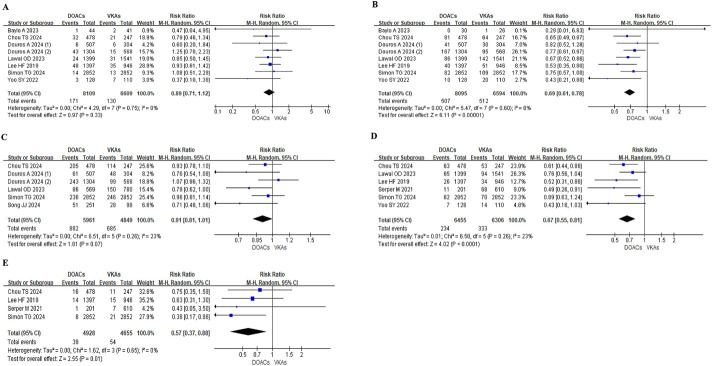
Meta-analysis of outcomes in patients with liver cirrhosis. DOAC, direct oral anticoagulant; VKA, vitamin K antagonist; IS, ischemic stroke; SE, systemic embolism; RR, risk ratio; CI, confidence interval. **(A)**. IS/SE **(B)**. Major bleeding **(C)**. All-cause mortality **(D)**. Gastrointestinal bleeding **(E)**. Intracranial bleeding.

In the subgroup of patients with liver cirrhosis and AF, DOACs similarly demonstrated a significantly lower risk of major bleeding (RR: 0.69, 95% CI: 0.61–0.78; P < 0.0001), GI bleeding (RR: 0.67, 95% CI: 0.55–0.81; P < 0.0001), and intracranial bleeding (RR: 0.57, 95% CI: 0.37–0.88; P = 0.01) compared to VKAs. However, the two groups did not differ significantly in IS/SE (RR: 0.89, 95% CI: 0.71–1.12; P = 0.33) or all-cause mortality (RR: 0.91, 95% CI: 0.81–1.01; P = 0.07) (Figure 3).

### 3.5 Subgroup analysis

Subgroup analyses were performed based on geographic region, follow-up duration, and specific drug selection.

**FIGURE 4:**
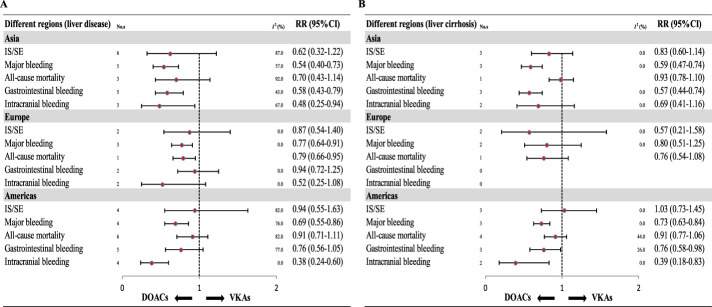
Subgroup analysis of data from different regions. DOAC, direct oral anticoagulant; VKA, vitamin K antagonist; IS, ischemic stroke; SE, systemic embolism; RR, risk ratio; CI, confidence interval. **(A)**. Liver disease **(B)**. Liver cirrhosis.

Among patients with AF and liver disease, DOACs consistently demonstrated a lower risk of major bleeding than VKAs across all regions (all P < 0.05), whereas the risk of IS/SE was similar between the two groups (all P > 0.05) (Figure 4A). In patients with liver cirrhosis, DOACs were associated with significantly reduced risks of major bleeding in both American (RR: 0.73, 95% CI: 0.63–0.84; P < 0.0001) and Asian populations (RR: 0.59, 95% CI: 0.47–0.74; P < 0.0001). However, no significant differences were observed in the risk of IS/SE between these regions (all P > 0.05). In contrast, among European patients with cirrhosis, DOACs and VKAs were associated with similar risks of major bleeding (RR: 0.80, 95% CI: 0.51–1.25, P = 0.32) and IS/SE (RR: 0.57, 95% CI: 0.21–1.58, P = 0.28) (Figure 4B).

**FIGURE 5:**
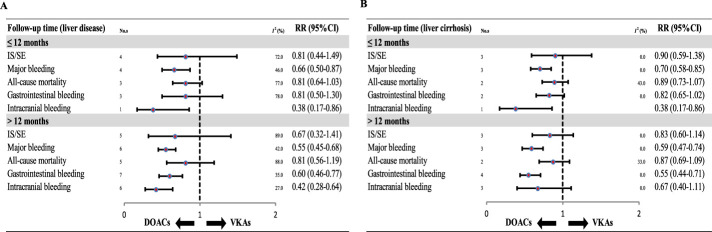
Subgroup analysis of data from different follow-up durations. DOAC, direct oral anticoagulant; VKA, vitamin K antagonist; IS, ischemic stroke; SE, systemic embolism; RR, risk ratio; CI, confidence interval. **(A)**. Liver disease **(B)**. Liver cirrhosis.

When stratified by follow-up time, in patients with AF and liver disease or cirrhosis, DOACs were associated with significantly lower risks of major bleeding and intracranial bleeding than VKAs during follow-up periods of less than 1 year (all P < 0.05). However, there were no statistically significant differences in GI bleeding, IS/SE, or all-cause mortality between the two groups in the short-term follow-up subgroup (all P > 0.05). In contrast, when the follow-up exceeded 1 year, DOACs were associated with significantly reduced risks of GI bleeding in both patients with liver disease (RR: 0.60, 95% CI: 0.46–0.77; P < 0.0001) and cirrhosis (RR: 0.55, 95% CI: 0.44–0.71; P < 0.0001) (Figure 5).

In a direct comparison between DOAC types, patients with AF and liver disease treated with apixaban had significantly lower risks of major bleeding (RR: 1.35, 95% CI: 1.16–1.56, P < 0.0001) and GI bleeding (RR: 1.40, 95% CI: 1.09–1.80, P = 0.0006) than those treated with rivaroxaban. No significant differences were observed in the other outcomes between the two drugs (all P > 0.05). Similarly, apixaban and rivaroxaban yielded comparable outcomes across all endpoints (all P > 0.05) in patients with cirrhosis.

**FIGURE 6:**
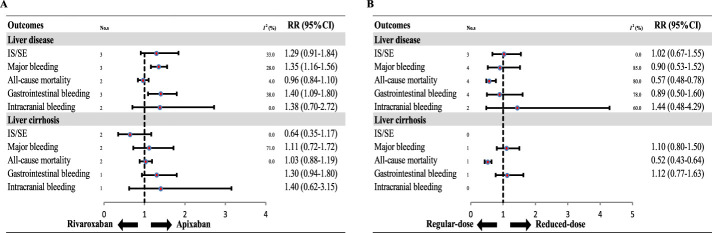
Subgroup analysis of data for direct oral anticoagulants (DOACs) (A. Rivaroxaban group vs Apixaban group; B. Regular-dose group vs Reduced-dose group). IS, ischemic stroke; SE, systemic embolism; ICH, intracranial hemorrhage; GI, Gastrointestinal; RR, risk ratio; CI, confidence interval. **(A)**. Rivaroxaban vs. Apixaban **(B)**. Regular-dose vs. Reduced-dose.

Lastly, when comparing standard-dose and low-dose DOAC regimens, patients with liver disease (RR: 0.57, 95% CI: 0.48–0.78; P = 0.0004) or cirrhosis (RR: 0.52, 95% CI: 0.43–0.64; P < 0.0001) who received standard-dose DOACs had a significantly lower risk of all-cause mortality. However, there were no statistically significant differences between the dosing groups for the other outcomes (all P > 0.05) (Figure 6).

### 3.6 Sensitivity analysis

Sensitivity analyses were performed to assess the robustness of the pooled results. The sequential exclusion of individual studies showed consistent findings across all outcomes, except for all-cause mortality in patients with AF and liver disease, which exhibited minor variability (Supplementary Tables S6 and S7). Further sensitivity analysis, excluding studies with a high risk of bias, revealed no significant changes in the results (Supplementary Tables S8 and S9), reinforcing the overall stability of the findings.

Additionally, when HR/aHR were used instead of RR, the incidence of all-cause mortality remained significantly lower in the DOACs than that in the VKAs among patients with AF and cirrhosis (HR: 0.81, 95% CI: 0.71–0.91; aHR: 0.82, 95% CI: 0.72–0.95). For all other outcomes, the results remained consistent when analyzed using either the RR or HR/aHR (Supplementary Figures S3 and S4). These findings suggest that the conclusions of this meta-analysis are robust and are not substantially influenced by individual studies or methodological differences. Composite outcomes (IS/SE, major bleeding and all-cause death) were analyzed separately for patients with liver disease (RR: 0.60, 95% CI: 0.43–0.85) and cirrhosis (RR: 0.68, 95% CI: 0.59–0.77), and the results indicated that DOACs were significantly more effective than VKAs.

### 3.7 Publication bias

Publication bias was assessed using funnel plots for primary clinical outcomes. These plots demonstrated visual symmetry, suggesting a low likelihood of publication bias. Additionally, Egger’s test did not indicate significant small study effects, further supporting the absence of publication bias in this meta-analysis (Supplementary Figures S5 and S6).

## 4 Discussion

For patients with deep vein thrombosis (DVT)/pulmonary embolism (PE) and liver disease or cirrhosis with portal vein thrombosis (PVT), relevant guidelines recommend DOACs to prevent thrombosis (European Association for the Study of the Liver, 2022; Northup et al., 2021). However, AF management guidelines do not provide specific recommendations for oral anticoagulants (OACs) selection in patients with liver disease (Joglar et al., 2023). Although high-quality RCTs are lacking, recent large-scale real-world data provide evidence supporting OACs selection. Previous meta-analyses suggest that DOACs and VKAs have comparable effectiveness in preventing thromboembolism, with DOACs associated with lower bleeding risk (Chen et al., 2022; Zhao et al., 2023; Li et al., 2023), but there is also a meta-analysis indicating that the IS/SE risk of DOACs is lower than that of VKAs (Huang et al., 2021). Therefore, the results of different meta-analyses are controversial. These earlier analyses often included small sample sizes and primarily focused on comparisons between DOACs and VKAs without exploring heterogeneity across patient subgroups. With the emergence of real-world data, updated meta-analyses are necessary to reflect broader populations and nuanced treatment considerations (Douros et al., 2024; Simon et al., 2024; Lawal et al., 2023). To address these research gaps, our study incorporated recent evidence and performed subgroup analyses by region and follow-up duration. We also evaluated the effectiveness and safety of different DOAC types and dosing regimens, contributing to a more refined understanding of treatment options.

Our findings showed that, in patients with liver disease and AF, DOACs were superior to VKAs in reducing major bleeding, intracranial bleeding, GI bleeding, and all-cause mortality. No significant differences were observed between groups in terms of IS/SE. These results are consistent with studies in patients with AF without liver disease, reinforcing the notion that DOACs offer a more favorable net clinical benefit than warfarin (Carnicelli et al., 2022). Most retrospective studies lack detailed clinical data on liver disease severity, including ascites, hepatic encephalopathy. And the ischemia and bleeding scores of patients with AF. Additionally, the absence of international normalized ratio (INR) data prevents assessment of the anticoagulant effect of VKAs. These limitations likely contributed to the high heterogeneity observed in the results.

In the United States, the use of DOACs in cirrhotic patients with AF increased substantially between 2012 and 2019 (from 18.7% to 77.6%) (Simon et al., 2023; Pereira Portela et al., 2024). This trend has outpaced the quality of available evidence, highlighting a disconnect between practice and data. In our cirrhosis subgroup analysis, the risks of major, GI, and intracranial bleeding were significantly lower with DOACs than with VKAs, whereas no significant differences were found in IS/SE or all-cause mortality. Nisly SA et al. reported no significant difference between DOACs and traditional anticoagulation in patients with cirrhosis (Nisly et al., 2021), while recent large-scale retrospective studies suggest that DOACs are safer. In our updated meta-analysis, DOACs demonstrated greater safety than VKAs, consistent with the findings of Miranda Maria et al. (2025). Furthermore, the present study confirmed that DOACs outperform VKAs in terms of composite effectiveness and safety outcomes.

Ethnic and regional variations may influence both liver disease etiology and thromboembolic risk. For example, thromboembolic events are more frequent in Asian populations than in non-Asian populations (Romiti et al., 2023). Moreover, alcoholic liver disease and nonalcoholic fatty liver disease are more prevalent in Europe and the Americas, whereas viral hepatitis predominates in Asia (Shepard et al., 2005; Blachier et al., 2013; Younossi et al., 2023). Our regional subgroup analysis demonstrated that the safety profile of DOACs is consistently better than that of VKAs in North America, Asia, and Europe. However, in European patients with cirrhosis, DOACs and VKAs showed comparable effectiveness and safety, although this conclusion was based on a limited number of studies and requires further validation. DOACs are particularly effective in reducing GI bleeding in Asian patients and intracranial bleeding in American patients. Since AF patients with liver disease often require long-term anticoagulation, we also analyzed outcomes according to follow-up duration. When follow-up was < 1 year, GI bleeding risk was similar between the groups; however, with follow-up beyond 1 year, DOACs significantly outperformed VKAs in reducing GI bleeding. These findings suggest that DOACs offer sustained benefits.

All oral anticoagulants undergo hepatic metabolism to varying extents (Speed et al., 2023), which raises concerns about increased drug exposure and bleeding risk in patients with liver impairment. To mitigate this, clinicians often reduce DOAC doses, although this may compromise their effectiveness. Our analysis found no significant difference in thrombotic or bleeding risks between low- and regular-dose DOACs. However, regular-dose DOACs are associated with lower all-cause mortality, suggesting a potential clinical advantage. This finding aligns with previous research (Chong et al., 2021; Cho et al., 2019), although it is important to consider that patients receiving lower doses tend to be older and have more comorbidities, which may underestimate the true benefits of standard dosing.

Different DOACs have demonstrated varied clinical outcomes (Ray et al., 2021; Dawwas et al., 2022). However, the optimal DOACs for patients with liver disease or cirrhosis remains unclear. Our results suggest that apixaban may be safer than rivaroxaban, with lower risks of major and GI bleeding, while both agents showed comparable risks of IS/SE, intracranial bleeding, and all-cause mortality. Based on pharmacokinetic studies, Frost et al. reported similar apixaban exposure between patients with mild-to-moderate hepatic dysfunction (Child-Pugh A/B) and healthy controls (Frost et al., 2021), whereas Kubitza et al. found that rivaroxaban exposure in patients with moderate hepatic impairment was more than twice that observed in patients with mild impairment or normal liver function (Kubitza et al., 2013). In addition, multiple studies have reported a higher risk of GI bleeding with rivaroxaban in patients with AF without liver disease (Ingason et al., 2021; Ray et al., 2021). These results suggest a lower risk of bleeding with apixaban in the clinical setting. Nonetheless, apixaban and rivaroxaban have demonstrated similar effectiveness and safety in patients with cirrhosis. This may be attributed to the higher biliary excretion of apixaban than that of rivaroxaban, resulting in greater hepatic accumulation and potential toxicity in severe liver dysfunction (Douros et al., 2024; Graff and Harder, 2013). This study updates the meta-analysis on the effectiveness and safety of DOACs in patients with liver disease and AF, offering reference for the advancement of guidelines or consensus. In addition, our results provide accurate suggestions for clinical administration in DOACs selection. Future research should prioritize personalized anticoagulation strategies based on liver disease severity, bleeding/thrombosis risk, and drug interactions. Further investigation into other DOACs, such as dabigatran and edoxaban, is also needed. Multi-center RCTs are essential to strengthen the current evidence base.

Our study has several limitations. First, most of the included studies were observational, RCTs are required to confirm these findings. Second, despite the use of multivariable adjustment methods, residual confounding factors cannot be ruled out. Notably, data on concomitant antiplatelet therapy, such as aspirin or P2Y12 inhibitors, which could influence bleeding risk, are often unavailable. Third, the severity of liver disease (e.g., Child–Turcotte–Pugh classification) has not been consistently reported, limiting stratified analyses. Fourth, adherence to long-term anticoagulant therapy and attainment of INR targets with VKA treatment could not be assessed in most studies (e.g., Time in Therapeutic Range (TTR) for patients treated with VKAs). Fifth, due to the limitation of the included literature, some secondary outcomes, including readmission risk, were not assessed. Finally, due to limited data, we were unable to evaluate dabigatran or edoxaban, and comparisons among DOACs were restricted to apixaban and rivaroxaban.

## 5 Conclusion

In patients with liver disease, DOACs were as effective as VKAs and demonstrated superior safety, including those with cirrhosis. DOACs consistently showed better outcomes across regions, except in European patients with cirrhosis, in whom no difference was observed. A longer follow-up was associated with a reduced risk of GI bleeding with DOACs. Although thrombotic and bleeding risks were similar between dosing regimens, Regular-dose DOACs reduced all-cause mortality. Apixaban has a better safety profile than rivaroxaban in liver disease, supporting its preferential use in individualized anticoagulation strategies. However, there was no difference in the effectiveness and safety of rivaroxaban and apixaban in patients with cirrhosis.

## Data Availability

The original contributions presented in the study are included in the article/Supplementary Material, further inquiries can be directed to the corresponding author.
