# Supplementary material for: Effectiveness and safety of direct oral anticoagulants versus vitamin K antagonists in atrial fibrillation patients with liver disease: a systematic review and meta-analysis
Source: Front Pharmacol. 2025 Jul 14;16:1620394. doi: 10.3389/fphar.2025.1620394 (PMC12301215; doi:10.3389/fphar.2025.1620394)
Supplement: Supplementary file 1 [file Supplementaryfile1.docx]

SUPPLEMENTAL MATERIAL

Title: Effectiveness and Safety of Direct Oral Anticoagulants Versus Vitamin K Antagonists in Atrial Fibrillation Patients with Liver Disease: A Systematic Review and Meta-analysis

**Contents**

Supplementary contents 1

**Tables**

[Supplementary Table 1. PRISMA 2020 Checklist 3](#_Toc2680868)

[Supplementary Table 2. Search strategy used in November, 2024 8](#_Toc2680868)

[Supplementary Table 3. Baseline characteristics of the included in studies 13](#_Toc2680868)

[Supplementary Table 4. GRADE Evidence Profile of Outcomes (liver disease) 17](#_Toc2680869)

Supplementary Table 5. GRADE Evidence Profile of Outcomes (liver cirrhosis) 19

[Supplementary Table 6. Sensitivity analysis by removing each item one by one (liver disease) 21](#_Toc2680871)

[Supplementary Table 7. Sensitivity analysis by removing each item one by one (liver cirrhosis) 22](#_Toc2680872)

[Supplementary Table 8. Sensitivity analysis after excluding low-quality studies (liver disease) 23](#_Toc2680872)

[**S**upplementary Table 9**.** Sensitivity analysis after excluding low-quality studies (liver cirrhosis) 24](#_Toc2680872)

**Figures**

[Supplementary Figure 1. Quality Assessment of the Included Studies Using ROB 25](#_Toc2680879)

[Supplementary Figure 2. Quality Assessment of the Included Studies Using ROBINS-I 26](#_Toc2680880)

[Supplementary Figure 3. Sensitivity analysis of outcomes between DOACs and VKAs (using HR as the statistical indicator) 27](#_Toc2680881)

[Supplementary Figure 4. Sensitivity analysis of outcomes between DOACs and VKAs (using aHR as the statistical indicator) 28](#_Toc2680881)

[Supplementary Figure 5. Funnel plot of outcomes in patients with AF and liver disease 29](#_Toc2680882)

[Supplementary Figure 6. Funnel plot of outcomes in patients with AF and liver cirrhosis 30](#_Toc2680883)

**Supplementary Table 1** PRISMA 2020 Checklist

| **Section and Topic** | **Item #** | **Checklist item** | **Location where item is reported** |
| --- | --- | --- | --- |
| **TITLE** | | | **PAGE** |
| Title | 1 | Identify the report as a systematic review. | 1 |
| **ABSTRACT** | | |  |
| Abstract | 2 | See the PRISMA 2020 for Abstracts checklist. | 1 |
| **INTRODUCTION** | | |  |
| Rationale | 3 | Describe the rationale for the review in the context of existing knowledge. | 2 |
| Objectives | 4 | Provide an explicit statement of the objective(s) or question(s) the review addresses. | 2 |
| **METHODS** | | |  |
| Eligibility criteria | 5 | Specify the inclusion and exclusion criteria for the review and how studies were grouped for the syntheses. | 2 |
| Information sources | 6 | Specify all databases, registers, websites, organisations, reference lists and other sources searched or consulted to identify studies. Specify the date when each source was last searched or consulted. | 2 |
| Search strategy | 7 | Present the full search strategies for all databases, registers and websites, including any filters and limits used. | 2 |
| Selection process | 8 | Specify the methods used to decide whether a study met the inclusion criteria of the review, including how many reviewers screened each record and each report retrieved, whether they worked independently, and if applicable, details of automation tools used in the process. | 2 |
| Data collection process | 9 | Specify the methods used to collect data from reports, including how many reviewers collected data from each report, whether they worked independently, any processes for obtaining or confirming data from study investigators, and if applicable, details of automation tools used in the process. | 3 |
| Data items | 10a | List and define all outcomes for which data were sought. Specify whether all results that were compatible with each outcome domain in each study were sought (e.g. for all measures, time points, analyses), and if not, the methods used to decide which results to collect. | 3 |
|  | 10b | List and define all other variables for which data were sought (e.g. participant and intervention characteristics, funding sources). Describe any assumptions made about any missing or unclear information. | 3 |
| Study risk of bias assessment | 11 | Specify the methods used to assess risk of bias in the included studies, including details of the tool(s) used, how many reviewers assessed each study and whether they worked independently, and if applicable, details of automation tools used in the process. | 3 |
| Effect measures | 12 | Specify for each outcome the effect measure(s) (e.g. risk ratio, mean difference) used in the synthesis or presentation of results. | 3 |
| Synthesis methods | 13a | Describe the processes used to decide which studies were eligible for each synthesis (e.g. tabulating the study intervention characteristics and comparing against the planned groups for each synthesis (item #5)). | 3 |
|  | 13b | Describe any methods required to prepare the data for presentation or synthesis, such as handling of missing summary statistics, or data conversions. | 3 |
|  | 13c | Describe any methods used to tabulate or visually display results of individual studies and syntheses. | 3 |
|  | 13d | Describe any methods used to synthesize results and provide a rationale for the choice(s). If meta-analysis was performed, describe the model(s), method(s) to identify the presence and extent of statistical heterogeneity, and software package(s) used. | 3 |
|  | 13e | Describe any methods used to explore possible causes of heterogeneity among study results (e.g. subgroup analysis, meta-regression). | 3 |
|  | 13f | Describe any sensitivity analyses conducted to assess robustness of the synthesized results. | 3 |
| Reporting bias assessment | 14 | Describe any methods used to assess risk of bias due to missing results in a synthesis (arising from reporting biases). | 3 |
| Certainty assessment | 15 | Describe any methods used to assess certainty (or confidence) in the body of evidence for an outcome. | 3 |
| **RESULTS** | | |  |
| Study selection | 16a | Describe the results of the search and selection process, from the number of records identified in the search to the number of studies included in the review, ideally using a flow diagram. | 3, Figure 1 |
|  | 16b | Cite studies that might appear to meet the inclusion criteria, but which were excluded, and explain why they were excluded. | Figure 1 |
| Study characteristics | 17 | Cite each included study and present its characteristics. | 3, Table 1, Supplementary Table 3 |
| Risk of bias in studies | 18 | Present assessments of risk of bias for each included study. | 3 |
| Results of individual studies | 19 | For all outcomes, present, for each study: (a) summary statistics for each group (where appropriate) and (b) an effect estimate and its precision (e.g. confidence/credible interval), ideally using structured tables or plots. | 4,6 |
| Results of syntheses | 20a | For each synthesis, briefly summarise the characteristics and risk of bias among contributing studies. | 4,6 |
|  | 20b | Present results of all statistical syntheses conducted. If meta-analysis was done, present for each the summary estimate and its precision (e.g. confidence/credible interval) and measures of statistical heterogeneity. If comparing groups, describe the direction of the effect. | 4,6 |
|  | 20c | Present results of all investigations of possible causes of heterogeneity among study results. | 6 |
|  | 20d | Present results of all sensitivity analyses conducted to assess the robustness of the synthesized results. | 6 |
| Reporting biases | 21 | Present assessments of risk of bias due to missing results (arising from reporting biases) for each synthesis assessed. | 7 |
| Certainty of evidence | 22 | Present assessments of certainty (or confidence) in the body of evidence for each outcome assessed. | 3, Supplementary Table 4-5 |
| **DISCUSSION** | | |  |
| Discussion | 23a | Provide a general interpretation of the results in the context of other evidence. | 7-8 |
|  | 23b | Discuss any limitations of the evidence included in the review. | 8-9 |
|  | 23c | Discuss any limitations of the review processes used. | 9 |
|  | 23d | Discuss implications of the results for practice, policy, and future research. | 9 |
| **OTHER INFORMATION** | | |  |
| Registration and protocol | 24a | Provide registration information for the review, including register name and registration number, or state that the review was not registered. | 2 |
|  | 24b | Indicate where the review protocol can be accessed, or state that a protocol was not prepared. | 2 |
|  | 24c | Describe and explain any amendments to information provided at registration or in the protocol. | N/A |
| Support | 25 | Describe sources of financial or non-financial support for the review, and the role of the funders or sponsors in the review. | 10 |
| Competing interests | 26 | Declare any competing interests of review authors. | 10 |
| Availability of data, code and other materials | 27 | Report which of the following are publicly available and where they can be found: template data collection forms; data extracted from included studies; data used for all analyses; analytic code; any other materials used in the review. | N/A |

**Supplementary Table 2** Search strategy used in November, 2024

| **Literature databases** | **Search items** | **Items found** |
| --- | --- | --- |
| PUBMED | “atrial fibrillation”[MeSH Terms] OR “atrial fibrillation”[Title/Abstract] OR “non-valvular atrial fibrillation”[MeSH Terms] OR “non-valvular atrial fibrillation”[Title/Abstract] OR “AF” [MeSH Terms] OR “AF”[Title/Abstract] OR “NVAF”[MeSH Terms] OR “NVAF”[Title/Abstract]  **AND**  “liver disease”[MeSH Terms] OR “liver disease”[Title/Abstract] OR “impaired liver function”[MeSH Terms] OR “impaired liver function”[Title/Abstract] OR “hepatic disease”[MeSH Terms] OR “hepatic disease”[Title/Abstract] OR “cirrhosis”[MeSH Terms] OR “cirrhosis”[Title/Abstract] OR “cirrhotic”[MeSH Terms] OR “cirrhotic”[Title/Abstract]  **AND**  “DOAC”[MeSH Terms] OR “DOAC”[Title/Abstract] OR “NOAC”[MeSH Terms] OR “NOAC”[Title/Abstract] OR “direct oral anticoagulant”[MeSH Terms] OR “direct oral anticoagulant”[Title/Abstract] OR “non-vitamin K antagonist oral anticoagulant”[MeSH Terms] OR “non-vitamin K antagonist oral anticoagulant”[Title/Abstract] OR “rivaroxaban”[MeSH Terms] OR “rivaroxaban”[Title/Abstract] OR “apixaban”[MeSH Terms] OR “apixaban”[Title/Abstract] OR “dabigatran”[MeSH Terms] OR “dabigatran”[Title/Abstract] OR “edoxaban”[MeSH Terms] OR “edoxaban”[Title/Abstract]  **AND**  “VKA”[MeSH Terms] OR “VKA”[Title/Abstract] OR “vitamin K antagonist”[MeSH Terms] OR “vitamin K antagonist”[Title/Abstract] OR “warfarin” [MeSH Terms] OR “warfarin”[Title/Abstract] | 795 |
| Medline | “atrial fibrillation”[MeSH Terms] OR  “atrial fibrillation”[Title/Abstract] OR “non-valvular atrial fibrillation”[MeSH Terms] OR “non-valvular atrial fibrillation”[Title/Abstract] OR “AF”[MeSH Terms] OR “AF”[Title/Abstract] OR “NVAF”[MeSH Terms] OR “NVAF”[Title/Abstract]  **AND**  “liver disease”[MeSH Terms] OR “liver disease”[Title/Abstract] OR “impaired liver function”[MeSH Terms] OR “impaired liver function”[Title/Abstract] OR “hepatic disease”[MeSH Terms] OR “hepatic disease”[Title/Abstract] OR “cirrhosis”[MeSH Terms] OR “cirrhosis”[Title/Abstract] OR “cirrhotic”[MeSH Terms] OR “cirrhotic”[Title/Abstract]  **AND**  “DOAC”[MeSH Terms] OR “DOAC”[Title/Abstract] OR “NOAC”[MeSH Terms] OR “NOAC”[Title/Abstract] OR “direct oral anticoagulant”[MeSH Terms] OR “direct oral anticoagulant”[Title/Abstract] OR “new oral anticoagulant”[MeSH Terms] OR “new oral anticoagulant”[Title/Abstract] OR “non-vitamin K antagonist oral anticoagulant”[MeSH Terms] OR “non-vitamin K antagonist oral anticoagulant”[Title/Abstract] OR “rivaroxaban”[MeSH Terms] OR “rivaroxaban”[Title/Abstract] OR “apixaban”[MeSH Terms] OR “apixaban”[Title/Abstract] OR “dabigatran”[MeSH Terms] OR “dabigatran”[Title/Abstract] OR “edoxaban”[MeSH Terms] OR “edoxaban”[Title/Abstract]  **AND**  “VKA”[MeSH Terms] OR “VKA”[Title/Abstract] OR “vitamin K antagonist”[MeSH Terms] OR “vitamin K antagonist”[Title/Abstract] OR “warfarin”[MeSH Terms] OR “warfarin”[Title/Abstract] | 761 |
| EMBASE | ‘atrial fibrillation’/exp OR ‘atrial fibrillation’:ti,ab,kw OR ‘non-valvular atrial fibrillation’/exp OR ‘non-valvular atrial fibrillation’: ti,ab,kw OR ‘AF’/exp OR ‘AF’: ti,ab,kw OR ‘NVAF’/exp OR ‘NVAF’: ti,ab,kw  **AND**  ‘liver disease’/exp OR ‘liver disease’:ti,ab,kw OR ‘impaired liver function’/exp OR ‘impaired liver function’: ti,ab,kw OR ‘hepatic disease’/exp OR ‘hepatic disease’: ti,ab,kw OR ‘cirrhosis’/exp OR ‘cirrhosis’: ti,ab,kw OR ‘cirrhotic’/exp OR ‘cirrhotic’: ti,ab,kw  **AND**  ‘DOAC’/exp OR ‘DOAC’:ti,ab,kw OR ‘NOAC’/exp OR ‘NOAC’: ti,ab,kw OR ‘direct oral anticoagulant’/exp OR ‘direct oral anticoagulant’: ti,ab,kw OR ‘new oral anticoagulant’/exp OR ‘new oral anticoagulant’: ti,ab,kw OR ‘non-vitamin K antagonist oral anticoagulant’/exp OR ‘non-vitamin K antagonist oral anticoagulant’: ti,ab,kw OR ‘rivaroxaban’/exp OR ‘rivaroxaban’: ti,ab,kw OR ‘apixaban’/exp OR ‘apixaban’: ti,ab,kw OR ‘dabigatran’/exp OR ‘dabigatran’: ti,ab,kw OR ‘edoxaban’/exp OR ‘edoxaban’: ti,ab,kw  **AND**  ‘VKA’/exp OR ‘VKA’:ti,ab,kw OR ‘vitamin K antagonist’/exp OR ‘vitamin K antagonist’: ti,ab,kw OR ‘warfarin’/exp OR ‘warfarin’: ti,ab,kw | 854 |
| COCHRANE | MeSH descriptor: [atrial fibrillation] OR atrial fibrillation: ti,ab,kw OR MeSH descriptor: [non-valvular atrial fibrillation] OR non-valvular atrial fibrillation: ti,ab,kw OR MeSH descriptor: [AF] OR AF: ti,ab,kw OR MeSH descriptor: [NVAF] OR NVAF: ti,ab,kw  **AND**  MeSH descriptor: [liver disease] OR liver disease: ti,ab,kw OR MeSH descriptor: [impaired liver function] OR impaired liver function: ti,ab,kw OR MeSH descriptor: [hepatic disease] OR hepatic disease: ti,ab,kw OR MeSH descriptor: [cirrhosis] OR cirrhosis: ti,ab,kw OR MeSH descriptor: [cirrhotic] OR cirrhotic: ti,ab,kw  **AND**  MeSH descriptor: [DOAC] OR DOAC: ti,ab,kw OR MeSH descriptor: [NOAC] OR NOAC: ti,ab,kw OR MeSH descriptor: [direct oral anticoagulant] OR direct oral anticoagulant: ti,ab,kw OR MeSH descriptor: [new oral anticoagulant] OR new oral anticoagulant: ti,ab,kw OR MeSH descriptor: [non-vitamin K antagonist oral anticoagulant] OR non-vitamin K antagonist oral anticoagulant: ti,ab,kw OR MeSH descriptor: [rivaroxaban] OR rivaroxaban: ti,ab,kw OR MeSH descriptor: [apixaban] OR apixaban: ti,ab,kw OR MeSH descriptor: [dabigatran] OR dabigatran: ti,ab,kw OR MeSH descriptor: [edoxaban] OR edoxaban: ti,ab,kw  **AND**  MeSH descriptor: [VKA] OR VKA: ti,ab,kw OR MeSH descriptor: [vitamin K antagonist] OR vitamin K antagonist: ti,ab,kw OR MeSH descriptor: [warfarin] OR warfarin: ti,ab,kw | 161 |
| Overall |  | 2571 |
| Duplication |  | 931 |

**Supplementary Table 3** Baseline characteristics of the included in studies

| **Author** | **Adjusted method** | | **Age (y)** | | | **Female (%)** | | | | **Hypertension (%)** | | **Child-Pugh score** | | **Diabetes (%)** | | |  |  |
| --- | --- | --- | --- | --- | --- | --- | --- | --- | --- | --- | --- | --- | --- | --- | --- | --- | --- | --- |
|  | **DOAC group** | **VKA**  **group** | **DOAC group** | | **VKA**  **group** | **DOAC group** | | | **VKA**  **group** | **DOAC group** | **VKA**  **group** | **DOAC group** | **VKA group** | **DOAC group** | | **VKA**  **group** |  |  |
| Chou TS 2024 | IPTW | | 72.2±11.3 | | 67.8±12.4 | 37.6 | | | 33.0 | 74.3 | 85.3 | Class A:55.4%; Class B:39.7%;  Class C:4.8%; | Class A:38.1%; Class B:54.7%;  Class C:7.3%; | 46.3 | | 48.0 |  |  |
| Douros A 2024 (UK) | IPTW | | 71.3±10.6 | | 71.4±10.4 | 41.86 | | | 40.13 | 79.07 | 78.90 | NA | NA | 42.12 | | 41.59 |  |  |
| Douros A 2024 ( Canada ) | IPTW | | 73.8±9.9 | | 73.6±9.7 | 40.76 | | | 42.17 | 88.74 | 88.98 | NA | NA | 46.12 | | 45.11 |  |  |
| Simon TG 2024 | Multivariable logistic regression models | | 73.7±7.2 | | 73.8±7.0 | 42.9 | | | 43.1 | 83.7 | 83.8 | NA | NA | 53.1 | | 52.2 |  |  |
| Song JJ 2024 | IPW-RA | | 64.4±14.5 | | | 39 | | | | NA | | NA | | NA | | |  |  |
| Lawal OD 2023 | IPTW | | 70.3±10.6 | | 70.3±10.4 | 42.4 | | | 42.8 | 87.6 | 87.7 | NA | NA | 45.6 | | 45.7 |  |  |
| Baylo A 2023 | NA | | 69.5 | | 69.0 | 30.0 | | | 42.3 | 73.3 | 61.5 | Class A:50%; Class B:50%; | Class A:34.6%; Class B:65.4%; | 6.7 | | 23.0 |  |  |
| Yoo SY 2022 | NA | | 70.4 | | 65.2 | 19.5 | | | 24.5 | 53.1 | 41.8 | Class A:78.1%; Class B:21.9%; | Class A:71.8%; Class B:28.2%; | 31.2 | | 36.4 |  |  |
| Serper M 2021 | PS | | 64.0±7.7 | | 64.6±7.5 | 99.5 | | | 98.2 | 96.0 | 98.2 | Class A:69.9%; Class B:29.5%;  Class C:0.65%; | Class A:91.5%; Class B:8.5%;  Class C:0%; | 55.2 | | 55.2 |  |  |
| Lee HF 2019 | PSSWs | | 72.80±11.05 | | 72.41±11.21 | 36.35 | | | 36.3 | 84.29 | 83.52 | NA | NA | 46.00 | | 44.89 |  |  |
| Qamar A 2019 | Random allocation | | 68.0±9.5 | 68.1±10.1, 69.2±9.4 | | | 30.6 | 36.6, 32.7 | | 34.0 | 33.7, 32.3 | NA | NA | 35.9 | 30.5, 33.5 | | | |
| Lee SR 2019 | PSM | | 67.9±10.2 | | 68.1±10.8 | 38.2 | | | 38.8 | 70.5 | 71.1 | NA | NA | 26.4 | 27.2 | | |  |
| Goriacko P 2018 | NA | | 66 | | 65 | 42.7 | | | 40.5 | NA | NA | Class A:64.0%; Class B:34.7%;  Class C:1.3%; | Class A:35.4%; Class B:58.9%;  Class C:5.7%; | NA | NA | | |  |
| Pastori D 2018 | NA | | 77.1±9.1 | | 72.7±8.9 | 44.1 | | | 48.3 | 88.9 | 84.2 | NA | NA | 24.6 | 22.7 | | |  |
| Wang CL 2018 | Adjusted the baseline differences | | 77.3±6.9 | | | 49.0 | | | | 69.2 | | NA | | 30.5 | | |  |  |

NA, not available; DOAC, direct oral anticoagulant; VKA, vitamin K antagonist; IPTW, inverse probability of treatment weighting; PSM, propensity score matching; PSSWs, propensity score-based stabilized weights; IPW-RA, inverse probability weighting with regression adjustment.

**Supplementary Table 3** Baseline characteristics of the included in studies (continued)

| **Author** | **Combined APT** | | | **NSAIDs** | | | **PPI/H2RA** | | | **CHA_2_DS_2_-VASc** | | | **HAS-BLED** | | |
| --- | --- | --- | --- | --- | --- | --- | --- | --- | --- | --- | --- | --- | --- | --- | --- |
|  | **DOAC group** | **VKA**  **group** | | **DOAC group** | **VKA**  **group** | | **DOAC group** | **VKA**  **group** | | **DOAC group** | **VKA**  **group** | | **DOAC group** | **VKA group** | |
| Chou TS 2024 | 43.0 | 28.6 | | 27.1 | 25.4 | | 32.1 | 35.2 | | 3.5±1.7 | 3.1±1.8 | | 3.9±1.3 | 3.6±1.4 | |
| Douros A 2024 (UK) | 44.75 | 44.10 | | 13.65 | 13.36 | | 54.82/6.09 | 54.91/6.07 | | NA | NA | | NA | NA | |
| Douros A 2024 ( Canada ) | 57.69 | 58.92 | | 15.45 | 15.40 | | 57.41/3.10 | 58.31/3.26 | | NA | NA | | NA | NA | |
| Simon TG 2024 | 11.4 | 11.6 | | 9.4 | 9.6 | | 46.2 | 45.8 | | 4.34±1.6 | 4.32±1.54 | | 2.62±0.9 | 2.62±0.88 | |
| Song JJ 2024 | 47 | | | NA | | | 52 | | | 2.8±1.3 | | | NA | | |
| Lawal OD 2023 | 10.0 | 10.0 | | NA | NA | | 41.0/7.6 | 41.8/7.7 | | 4.0±2.4 | 4.0±2.7 | | 4.1±1.7 | 4.1±1.9 | |
| Baylo A 2023 | 23.3 | 34.6 | | 16.67 | 27 | | 212.0 | 210.0 | | NA | NA | | NA | NA | |
| Yoo SY 2022 | 21.8 | 40.0 | | NS | NS | | 146 | 143 | | 2.6±1.3 | 1.9±1.4 | | 2.2±0.9 | 2.1±1.0 | |
| Serper M 2021 | 58.2 | 59.5 | | NS | NS | | NS | NS | | 1.9±0.9 | 2.1±1.1 | | NA | NA | |
| Lee HF 2019 | NA | NA | | 26.46 | 25.34 | | 21.89/39.92 | 22.69/40.62 | | 38.67 | 39.80 | | NA | NA | |
| Qamar A 2019 | 34.3 | | 33.3, 32.4 | NA | | NA | NA | | NA | 4.2±1.4 | | 4.2±1.5, 4.4±1.4 | 34.2% Score≥3 | | 32.0% Score≥3, 33.8% Score≥3 |
| Lee SR 2019 | NA | NA | | NA | NA | | NA | NA | | 3.4±1.9 | 3.4±1.6 | | NA | NA | |
| Goriacko P 2018 | 52 | 42.41 | | 25.5 | 10.1 | | NA | NA | | NA | NA | | NA | NA | |
| Pastori D 2018 | 10.8 | 11.3 | | NA | NA | | NA | NA | | 3.0±1.5 | 3.4±1.4 | | 1.8±1.0 | 1.5±0.8 | |
| Wang CL 2018 | 58.5 | | | 23.2 | | | NA | | | NA | | | NA | | |

NA, not available; DOAC, direct oral anticoagulant; VKA, vitamin K antagonist; PPI, proton pump inhibitor, APT, antiplatelet therapy; NSAIDs, non steroidal anti-inflammatory drugs; H2RA, H2 receptor antagonist; CHA2DS2-VASc, a score for assessing stroke risk in atrial fibrillation, including heart failure, hypertension, age, diabetes, prior stroke, vascular disease, and sex; HAS-BLED, a score to estimate bleeding risk in atrial fibrillation, considering hypertension, renal/liver function, stroke, bleeding history, labile INR, age, and substance use.

**Supplementary Table 4** GRADE Evidence Profile of Outcomes (liver disease)

**Summary of findings:**

| **Direct oral anticoagulants compared to Vitamin K antagonists for Atrial fibrillation and liver disease** | | | | | | |
| --- | --- | --- | --- | --- | --- | --- |
| **Patient or population:** Atrial fibrillation and liver disease  **Intervention:** Direct oral anticoagulants  **Comparison:** Vitamin K antagonists | | | | | | |
| Outcomes | **Anticipated absolute effects^*^** (95% CI) | | Relative effect (95% CI) | № of participants (studies) | Certainty of the evidence (GRADE) | Comments |
|  | **Risk with Vitamin K antagonists** | **Risk with Direct oral anticoagulants** |  |  |  |  |
| Ischemic stroke/systemic embolism | 29 per 1,000 | **22 per 1,000** (15 to 33) | **RR 0.77** (0.52 to 1.13) | 35939 (10 non-randomised studies) | ⨁◯◯◯ Very low^a,b^ | The use of DOACs not significantly reduce the risk of ischemic stroke/SE in AF with liver disease patients. |
| Major bleeding | 78 per 1,000 | **50 per 1,000** (43 to 59) | **RR 0.64** (0.55 to 0.75) | 38240 (11 non-randomised studies) | ⨁⨁◯◯ Low^a,c^ | The use of DOACs significantly reduces the risk of major bleeding in AF with liver disease patients. |
| All-cause death | 133 per 1,000 | **110 per 1,000** (93 to 130) | **RR 0.83** (0.70 to 0.98) | 33855 (9 non-randomised studies) | ⨁◯◯◯ Very low^a,b^ | The use of DOACs significantly reduces the risk of all-cause death in AF with liver disease patients. |
| Gastrointestinal bleeding | 45 per 1,000 | **33 per 1,000** (27 to 40) | **RR 0.72** (0.59 to 0.89) | 38995 (11 non-randomised studies) | ⨁⨁◯◯ Low^a,c^ | The use of DOACs significantly reduces the risk of gastrointestinal bleeding in AF with liver disease patients. |
| Intracranial bleeding | 12 per 1,000 | **5 per 1,000** (4 to 7) | **RR 0.42** (0.32 to 0.55) | 29819 (8 non-randomised studies) | ⨁⨁⨁⨁ High^a^ | The use of DOACs significantly reduces the risk of intracranial bleeding in AF with liver disease patients. |
| ***The risk in the intervention group** (and its 95% confidence interval) is based on the assumed risk in the comparison group and the **relative effect** of the intervention (and its 95% CI). **CI:** confidence interval; **RR:** risk ratio | | | | | | |
| **GRADE Working Group grades of evidence** **High certainty:** we are very confident that the true effect lies close to that of the estimate of the effect. **Moderate certainty:** we are moderately confident in the effect estimate: the true effect is likely to be close to the estimate of the effect, but there is a possibility that it is substantially different. **Low certainty:** our confidence in the effect estimate is limited: the true effect may be substantially different from the estimate of the effect. **Very low certainty:** we have very little confidence in the effect estimate: the true effect is likely to be substantially different from the estimate of effect. | | | | | | |

#### Explanations

a. Medium risk research has a higher weight and may have a certain impact on the results. After comprehensive evaluation, consider downgrading by one level.

b. I²＞80%, Indicating significant heterogeneity.

c. I²＞50%, Indicating significant heterogeneity.

**Supplementary Table 5** GRADE Evidence Profile of Outcomes (liver cirrhosis)

| **Summary of findings:** | | | | | | |
| --- | --- | --- | --- | --- | --- | --- |
| **Direct oral anticoagulants compared to Vitamin K antagonists for Atrial fibrillation and cirrhosis** | | | | | | |
| **Patient or population:** Atrial fibrillation and cirrhosis  **Intervention:** Direct oral anticoagulants  **Comparison:** Vitamin K antagonists | | | | | | |
| Outcomes | **Anticipated absolute effects^*^** (95% CI) | | Relative effect (95% CI) | № of participants (studies) | Certainty of the evidence (GRADE) | Comments |
|  | **Risk with Vitamin K antagonists** | **Risk with Direct oral anticoagulants** |  |  |  |  |
| Ischemic stroke/systemic embolism | 20 per 1,000 | **18 per 1,000** (14 to 22) | **RR 0.89** (0.71 to 1.12) | 14718 (7 non-randomised studies) | ⨁⨁⨁◯ Moderate^a^ | The use of DOACs not significantly reduce the risk of ischemic stroke/SE in AF with cirrhosis patients. |
| Major bleeding | 78 per 1,000 | **54 per 1,000** (47 to 61) | **RR 0.69** (0.61 to 0.78) | 14689 (7 non-randomised studies) | ⨁⨁⨁◯ Moderate^a^ | The use of DOACs significantly reduces the risk of major bleeding in AF with cirrhosis patients. |
| All-cause death | 141 per 1,000 | **130 per 1,000** (119 to 141) | **RR 0.92** (0.84 to 1.00) | 10810 (5 non-randomised studies) | ⨁⨁◯◯ Low^a,b^ | The use of DOACs not significantly reduce the risk of all-cause death in AF with cirrhosis patients. |
| Gastrointestinal bleeding | 53 per 1,000 | **36 per 1,000** (31 to 42) | **RR 0.68** (0.58 to 0.80) | 12761 (6 non-randomised studies) | ⨁⨁⨁◯ Moderate^a^ | The use of DOACs significantly reduces the risk of gastrointestinal bleeding in AF with cirrhosis patients. |
| Intracranial bleeding | 12 per 1,000 | **6 per 1,000** (4 to 10) | **RR 0.56** (0.36 to 0.85) | 9583 (4 non-randomised studies) | ⨁⨁◯◯ Low^a,c^ | The use of DOACs significantly reduces the risk of intracranial bleeding in AF with cirrhosis patients. |
| ***The risk in the intervention group** (and its 95% confidence interval) is based on the assumed risk in the comparison group and the **relative effect** of the intervention (and its 95% CI). **CI:** confidence interval; **RR:** risk ratio | | | | | | |
| **GRADE Working Group grades of evidence** **High certainty:** we are very confident that the true effect lies close to that of the estimate of the effect. **Moderate certainty:** we are moderately confident in the effect estimate: the true effect is likely to be close to the estimate of the effect, but there is a possibility that it is substantially different. **Low certainty:** our confidence in the effect estimate is limited: the true effect may be substantially different from the estimate of the effect. **Very low certainty:** we have very little confidence in the effect estimate: the true effect is likely to be substantially different from the estimate of effect. | | | | | | |

#### Explanations

a. Medium risk research has a higher weight and may have a certain impact on the results. After comprehensive evaluation, consider downgrading by one level.

b. The effect estimation only comes from five observational studies.

c. The effect estimation only comes from four observational studies.

**Supplemental Table 6** Sensitivity analysis by removing each item one by one (liver disease)

| **Omitted IS/SE** | **RR (95% CI)** | **Omitted major bleeding** | **RR (95% CI)** | **Omitted all-cause mortality** | **RR (95% CI)** | **Omitted GI bleeding** | **RR (95% CI)** | **Omitted**  **intracranial bleeding** | **RR (95% CI)** |
| --- | --- | --- | --- | --- | --- | --- | --- | --- | --- |
| Chou TS 2024 | 0.77 (0.50–1.18) | Chou TS 2024 | 0.64 (0.54–0.76) | Chou TS 2024 | 0.81 (0.67–0.98) | Chou TS 2024 | 0.74 (0.59–0.92) | Chou TS 2024 | 0.39 (0.30–0.52) |
| Douros A 2024 | 0.72 (0.45–1.15) | Douros A 2024 | 0.59 (0.50–0.68) | Douros A 2024 | 0.80 (0.65–0.98) | Douros A 2024 | 0.66 (0.53–0.82) | Douros A 2024 | 0.41 (0.29–0.58) |
| Simon TG 2024 | 0.75 (0.50–1.13) | Simon TG 2024 | 0.63 (0.53–0.75) | Simon TG 2024 | 0.81 (0.67–0.98) | Simon TG 2024 | 0.71 (0.56–0.88) | Simon TG 2024 | 0.44 (0.32–0.60) |
| Lawal OD 2023 | 0.82 (0.53–1.26) | Lawal OD 2023 | 0.66 (0.56–0.77) | Song JJ 2024 | 0.84 (0.70–1.00) | Lawal OD 2023 | 0.75 (0.60–0.93) | Serper M 2021 | 0.43 (0.32–0.58) |
| Baylo A 2023 | 0.78 (0.53–1.16) | Baylo A 2023 | 0.64 (0.55–0.75) | Lawal OD 2023 | 0.85 (0.70–1.02) | Yoo SY 2022 | 0.74 (0.60–0.91) | Lee HF 2019 | 0.40 (0.30–0.53) |
| Yoo SY 2022 | 0.80 (0.54–1.20) | Yoo SY 2022 | 0.65 (0.56–0.76) | Qamar A 2019 | 0.80 (0.67–0.95) | Serper M 2021 | 0.74 (0.60–0.92) | Qamar A 2019 | 0.44 (0.33–0.58) |
| Lee HF 2019 | 0.75 (0.49–1.16) | Lee HF 2019 | 0.65 (0.55–0.77) | Lee SR 2019 | 0.84 (0.71–1.00) | Lee HF 2019 | 0.74 (0.60–0.92) | Lee SR 2019 | 0.50 (0.36–0.68) |
| Qamar A 2019 | 0.72 (0.48–1.06) | Qamar A 2019 | 0.64 (0.54–0.75) | Goriacko P 2018 | 0.88 (0.77–1.01) | Qamar A 2019 | 0.70 (0.57–0.87) | Pastori D 2018 | 0.44 (0.32–0.59) |
| Lee SR 2019 | 0.89 (0.68–1.16) | Lee SR 2019 | 0.68 (0.60–0.78) | Wang CL 2018 | 0.83 (0.69–0.99) | Lee SR 2019 | 0.76 (0.62–0.93) |  |  |
| Wang CL 2018 | 0.73 (0.49–1.10) | Pastori D 2018 | 0.64 (0.54–0.76) |  |  | Pastori D 2018 | 0.71 (0.57–0.87) |  |  |
|  |  | Wang CL 2018 | 0.63 (0.54–0.74) |  |  | Wang CL 2018 | 0.70 (0.57–0.86) |  |  |

IS, ischemic stroke; SE, systemic embolism; GI, Gastrointestinal; RR, risk ratio; CI, confidence interval.

**Supplemental Table 7** Sensitivity analysis by removing each item one by one (liver cirrhosis)

| **Omitted IS/SE** | **RR (95% CI)** | **Omitted major bleeding** | **RR (95% CI)** | **Omitted all-cause mortality** | **RR (95% CI)** | **Omitted GI bleeding** | **RR (95% CI)** | **Omitted**  **intracranial bleeding** | **RR (95% CI)** |
| --- | --- | --- | --- | --- | --- | --- | --- | --- | --- |
| Chou TS 2024 | 0.92 (0.71–1.19) | Chou TS 2024 | 0.70 (0.61–0.79) | Chou TS 2024 | 0.89 (0.77–1.03) | Chou TS 2024 | 0.67 (0.52–0.87) | Chou TS 2024 | 0.50 (0.30–0.84) |
| Douros A 2024 | 0.85 (0.66–1.10) | Douros A 2024 | 0.65 (0.56–0.76) | Douros A 2024 | 0.89 (0.80–1.00) | Simon TG 2024 | 0.63 (0.52–0.76) | Simon TG 2024 | 0.67 (0.40–1.11) |
| Simon TG 2024 | 0.88 (0.69–1.11) | Simon TG 2024 | 0.67 (0.59–0.77) | Simon TG 2024 | 0.88 (0.77–1.01) | Lawal OD 2023 | 0.63 (0.49–0.81) | Serper M 2021 | 0.58 (0.37–0.90) |
| Lawal OD 2023 | 0.90 (0.70–1.16) | Lawal OD 2023 | 0.70 (0.61–0.80) | Song JJ 2024 | 0.92 (0.83–1.03) | Yoo SY 2022 | 0.68 (0.56–0.84) | Lee HF 2019 | 0.54 (0.32–0.92) |
| Baylo A 2023 | 0.90 (0.71–1.13) | Baylo A 2023 | 0.69 (0.61–0.78) | Lawal OD 2023 | 0.93 (0.84–1.04) | Serper M 2021 | 0.69 (0.56–0.85) |  |  |
| Yoo SY 2022 | 0.92 (0.73–1.16) | Yoo SY 2022 | 0.70 (0.62–0.79) |  |  | Lee HF 2019 | 0.69 (0.56–0.85) |  |  |
| Lee HF 2019 | 0.88 (0.67–1.15) | Lee HF 2019 | 0.71 (0.63–0.80) |  |  |  |  |  |  |

IS, ischemic stroke; SE, systemic embolism; GI, Gastrointestinal; RR, risk ratio; CI, confidence interval.

**Supplementary Table 8** Sensitivity analysis after excluding low-quality studies (liver disease)

| Variable | No.s | Effect | | |
| --- | --- | --- | --- | --- |
|  |  | RR (95%CI) | *P* value | *I²* (%) |
| IS/SE | 9 | 0.78（0.53-1.16） | 0.22 | 86 |
| Major bleeding | 9 | 0.64（0.54-0.76） | <0.0001* | 72 |
| All cause death | 8 | 0.83（0.69-0.99） | 0.04* | 88 |
| Intracranial bleeding | 6 | 0.44（0.32-0.61） | <0.0001* | 24 |
| GI bleeding | 9 | 0.76（0.58-0.90） | 0.004* | 71 |

RR, risk ratio; CI, confidence interval; IS, ischemic stroke; SE, systemic embolism; GI, Gastrointestinal.

**Supplementary Table 9** Sensitivity analysis after excluding low-quality studies (liver cirrhosis)

| Variable | No.s | Effect | | |
| --- | --- | --- | --- | --- |
|  |  | RR (95%CI) | *P* value | *I²* (%) |
| IS/SE | 6 | 0.90（0.71-1.13） | 0.36 | 0 |
| Major bleeding | 6 | 0.69（0.61-0.78） | <0.0001* | 0 |
| All cause death | 4 | 0.92（0.83-1.03） | 0.14 | 18 |
| Intracranial bleeding | 3 | 0.58（0.37-0.90） | 0.01* | 0 |
| GI bleeding | 5 | 0.69（0.56-0.85） | <0.0001* | 25 |

RR, risk ratio; CI, confidence interval; IS, ischemic stroke; SE, systemic embolism; GI, Gastrointestinal.

**
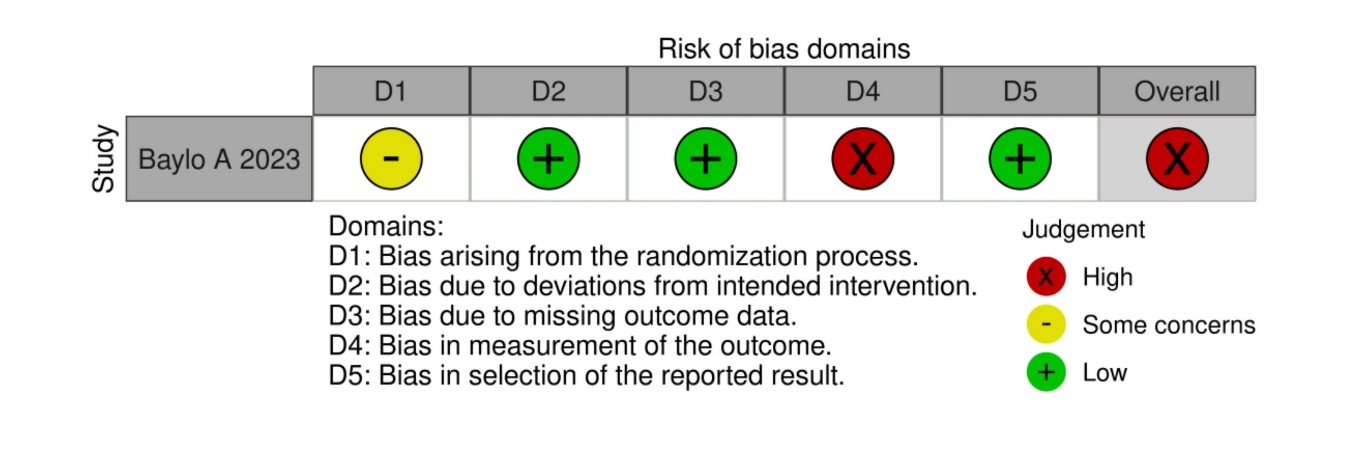
**

**Supplemental Figure 1** Quality Assessment of the Included Studies Using ROB.

**
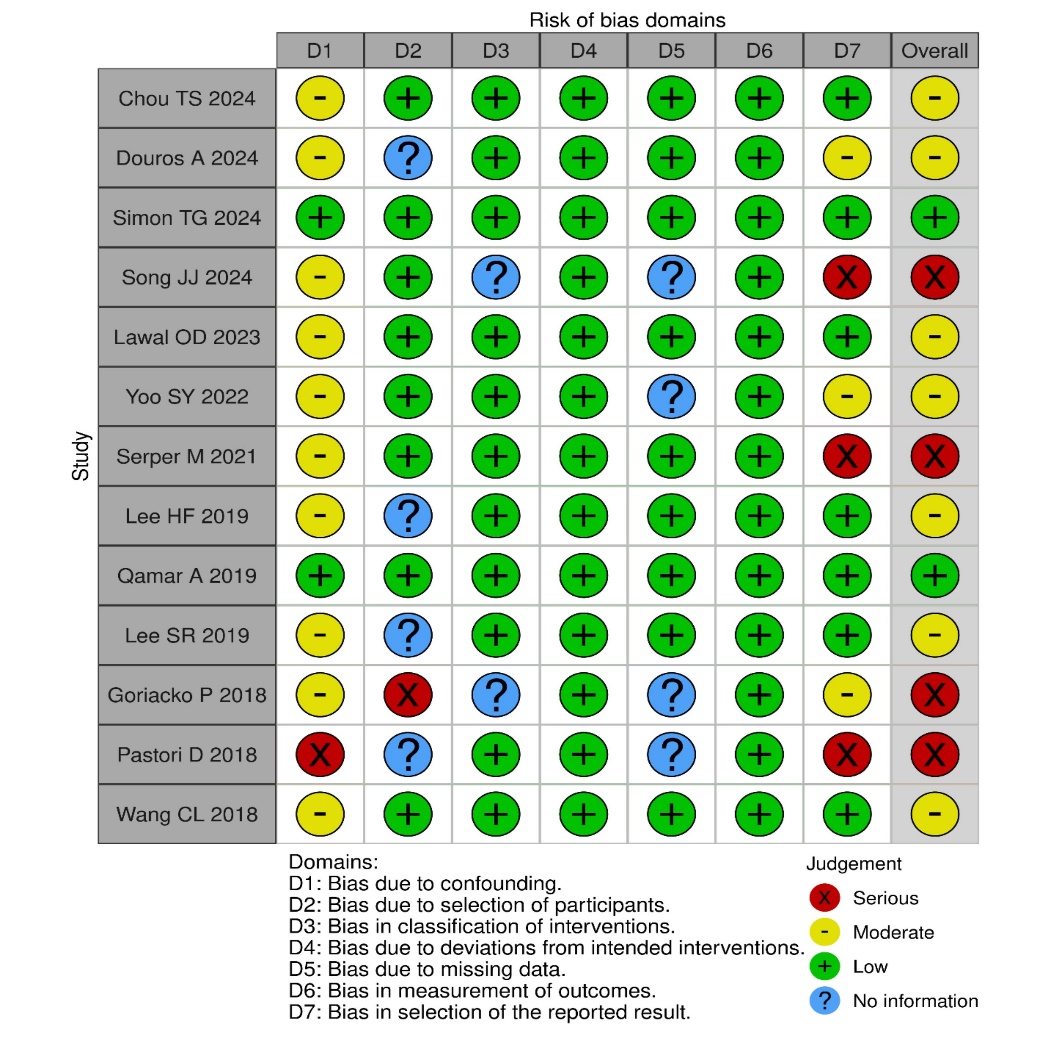
**

**Supplemental Figure 2** Quality Assessment of the Included Studies Using ROBINS-I.

**Supplementary Figure 3** Sensitivity analysis of outcomes between DOACs and VKAs (using HR as the statistical indicator). DOAC, direct oral anticoagulant; VKA, vitamin K antagonist; IS, ischemic stroke; SE, systemic embolism; HR, hazard ratios; CI, confidence interval.

**Supplementary Figure 4** Sensitivity analysis of outcomes between DOACs and VKAs (using aHR as the statistical indicator). DOAC, direct oral anticoagulant; VKA, vitamin K antagonist; IS, ischemic stroke; SE, systemic embolism; aHR, adjusted hazard ratios; CI, confidence interval.

P=0.452

P=0.286

P=0.989

P=0.938

P=0.660

**A**

**B**

**C**

**D**

**E**

**Supplemental Figure** **5** Funnel plot of outcomes in patients with AF and liver disease (A. IS/SE; B. Major bleeding; C. All-cause mortality; D. Gastrointestinal bleeding; E. Intracranial bleeding). AF, atrial fibrillation; IS, ischemic stroke; SE, systemic embolism.

P=0.153

P=0.106

P=0.082

P=0.687

P=0.134

**A**

**B**

**C**

**D**

**E**

**Supplemental Figure 6** Funnel plot of outcomes in patients with AF and liver cirrhosis (A. IS/SE; B. Major bleeding; C. All-cause mortality; D. Gastrointestinal bleeding; E. Intracranial bleeding). AF, atrial fibrillation; IS, ischemic stroke; SE, systemic embolism.
